# Demographic reporting across a decade of neuroimaging: a systematic review

**DOI:** 10.1007/s11682-022-00724-8

**Published:** 2022-09-17

**Authors:** Elijah Sterling, Hannah Pearl, Zexuan Liu, Jason W. Allen, Candace C. Fleischer

**Affiliations:** 1 College of Arts and Sciences, Emory University, Atlanta, GA USA; 2 School of Arts and Sciences, Tufts University, Medford, MA USA; 3 Department of Biomedical Engineering, Georgia Institute of Technology and Emory University, Atlanta, GA USA; 4 Department of Radiology and Imaging Sciences, Emory University School of Medicine, Atlanta, GA USA; 5 Department of Neurology, Emory University School of Medicine, Atlanta, GA USA

**Keywords:** Demographic reporting, NIH Revitalization Act of 1993, Neuroimaging, MRI

## Abstract

Diversity of participants in biomedical research with respect to race, ethnicity, and biological sex is crucial, particularly given differences in disease prevalence, recovery, and survival rates between demographic groups. The objective of this systematic review was to report on the demographics of neuroimaging studies using magnetic resonance imaging (MRI). The Web of Science database was used and data collection was performed between June 2021 to November 2021; all articles were reviewed independently by at least two researchers. Articles utilizing MR data acquired in the United States, with n ≥ 10 human subjects, and published between 2010–2020 were included. Non-primary research articles and those published in journals that did not meet a quality control check were excluded. Of the 408 studies meeting inclusion criteria, approximately 77% report sex, 10% report race, and 4% report ethnicity. Demographic reporting also varied as function of disease studied, participant age range, funding, and publisher. We anticipate quantitative data on the extent, or lack, of reporting will be necessary to ensure inclusion of diverse populations in biomedical research.

**Supplementary Information:**

The online version contains supplementary material available at 10.1007/s11682-022-00724-8.

Health disparities, disease presentation, and patient outcomes are influenced by a wide range of factors including sex, race, and ethnicity (Tang et al. 2022; Shakeshaft et al. 2022; Nahar et al. 2022; Tseng, et al. 2020). In biomedical and neuroimaging research, differences across demographic groups have been observed in brain structure, anatomy, morphometry, connectivity, development, and disease (Allouh et al. 2020; Bonkhoff et al. 2021; Chand et al. 2017; Delvecchio et al. 2021; Giedd et al. 2012; Hosseini et al. 2018; Liu et al. 2020). Despite documented differences between demographic groups, historical underrepresentation of minority and female participants has resulted in clinical and biomedical best practices informed by conclusions made using a narrow subset of the population (Brady et al. 2021; Sardar et al. 2014; Clayton and Collins 2014).

In the United States (U.S.), the National Institutes of Health (NIH) announced a policy in 1986 to increase the inclusion of women and minorities in clinical studies, motivated by initial reports and testimony demonstrating women and minorities had not been effectively integrated into clinical studies (https://www.gao.gov/products/t-hrd-90-38). The Office of Research on Women’s Health was established in 1990 by the NIH and advocated for policies mandating inclusion of women in clinical research (Clayton and Collins 2014). The subsequent NIH Revitalization Act of 1993 provides guidelines for including women and minorities in NIH-supported research. Since its implementation, however, a lack of reporting as well as a lack of diversity with respect to sex, race, and ethnicity of participants has persisted (Burchard 2015; Oh, et al. 2015; Reihl et al. 2022; Steinbrenner et al. 2022). For example, in clinical trials funded by the NIH National Cancer Institute between 1993–2013, less than 2% of studies reported demographics consistent with the requirements of the NIH Revitalization Act (Chen et al. 2014).

Both underrepresentation and underreporting are current challenges (Dotson and Duarte 2020; Raman et al. 2021). A review of cognitive neuroscience studies published between 2018–2019 revealed 14% of the 208 original research articles reported race or ethnicity (Dotson and Duarte 2020). Underrepresentation and underreporting of race and ethnicity has been observed in the study of numerous conditions including pulmonary disease (Burchard 2015), glioblastoma (Zhang et al. 2017), and autism spectrum disorder (Steinbrenner et al. 2022). For studies that do report demographics, underrepresentation of non-White participants is common. In a trial in pre-clinical Alzheimer’s disease of nearly 6,000 individuals between 2014–2017, ~ 86% of participants were White and recruitment efforts and methods differed as a function of race (Raman et al. 2021). While White participants were recruited broadly (e.g., local and national media, organizational referrals), Black, Asian, and Hispanic participants were primarily recruited via local site recruitment. Similarly, a review of clinical trials in neuro-oncology, including 662 studies from 2000–2019, also reported predominantly White participants (95%) (Reihl et al. 2022). It is important to note the true demographic distribution across these studies can often not be concluded given the overall lack of reporting.

In recent years, biomedical research has experienced an increase in neuroimaging studies utilizing magnetic resonance imaging (MRI), likely due, in part, to the increased use of MRI in diagnostic imaging (Smith-Bindman et al. 2009). The number of participants in MRI studies is also steadily increasing (Smith-Bindman et al. 2009; Szucs and Ioannidis 2020). Neuroimaging databases and efforts such as the NIH-supported Human Connectome Project (HCP) and the United Kingdom (UK) Biobank provide further evidence for the importance of MRI in neuroscience, particularly the availability of open-access repositories of multi-parametric MR data collected from healthy subjects (Isherwood et al. 2021; Eickhoff et al. 2016; Poldrack and Gorgolewski 2014; Madan 2021).

In light of the increasing use of MRI in biomedical and clinical research and given limited data on the demographics of participants included in neuroimaging studies, we conducted a systematic review to quantify the degree of demographic reporting as well as the reported diversity of human subjects participating in brain MR studies in the U.S. published from 2010–2020 (see Supplementary Methods). Of 3,458 articles meeting initial search criteria, 408 articles were included in the final analysis (Fig. 1). Of note, trends and summary statistics throughout are reported in whole number percentages for simplicity. All raw data collected from included articles are provided in Supplemental Table 1. Biological sex was reported in 315 (77% of all included articles), race in 41 (10%), and ethnicity in 17 (4%) included articles. A total of 49 articles (12% of the 408 articles included) utilized an existing database or publicly available dataset. Of these, 23 (47% of studies using a database) reported sex, 4 (8%) reported race, and 1 (2%) reported ethnicity of study participants. The most utilized database was the HCP (*n* = 13, 26% of studies using a database). We attribute the lack of reporting for studies using existing databases, in part, to the limited availability of some demographic information. In the HCP database, for example, sex of participants is available as open-access data but race, ethnicity, and age in years are restricted and cannot be published at the individual level. Below, we describe the results of our systematic review on reporting sex, race, and ethnicity, and discuss how these trends vary as a function of disease of focus, age of participants, NIH funding, and publisher in the context of prior research. Barriers to reporting and inclusion are discussed, and we conclude with recommendations for the future.

**Fig. 1:**
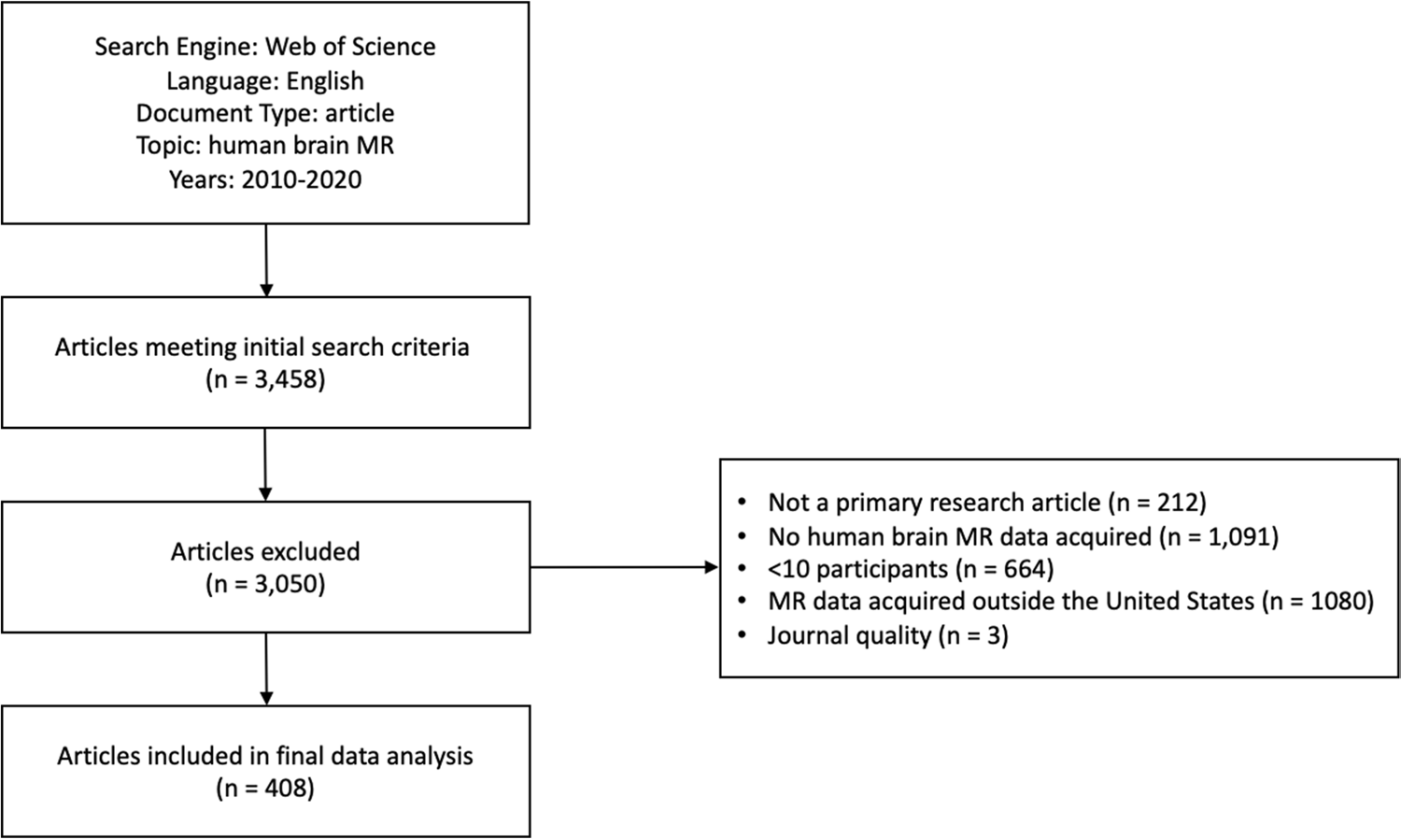
Flow chart for systematic review. A total of 408 articles published between 2010–2020 were included in our review and analysis. Articles were excluded in the order listed as many articles met multiple exclusion criteria. A summary of all included articles is provided in Supplementary Table 1 and detailed methods are described in Supplementary Methods

## Biological sex is largely reported and males and females are equally represented

**Fig. 2:**
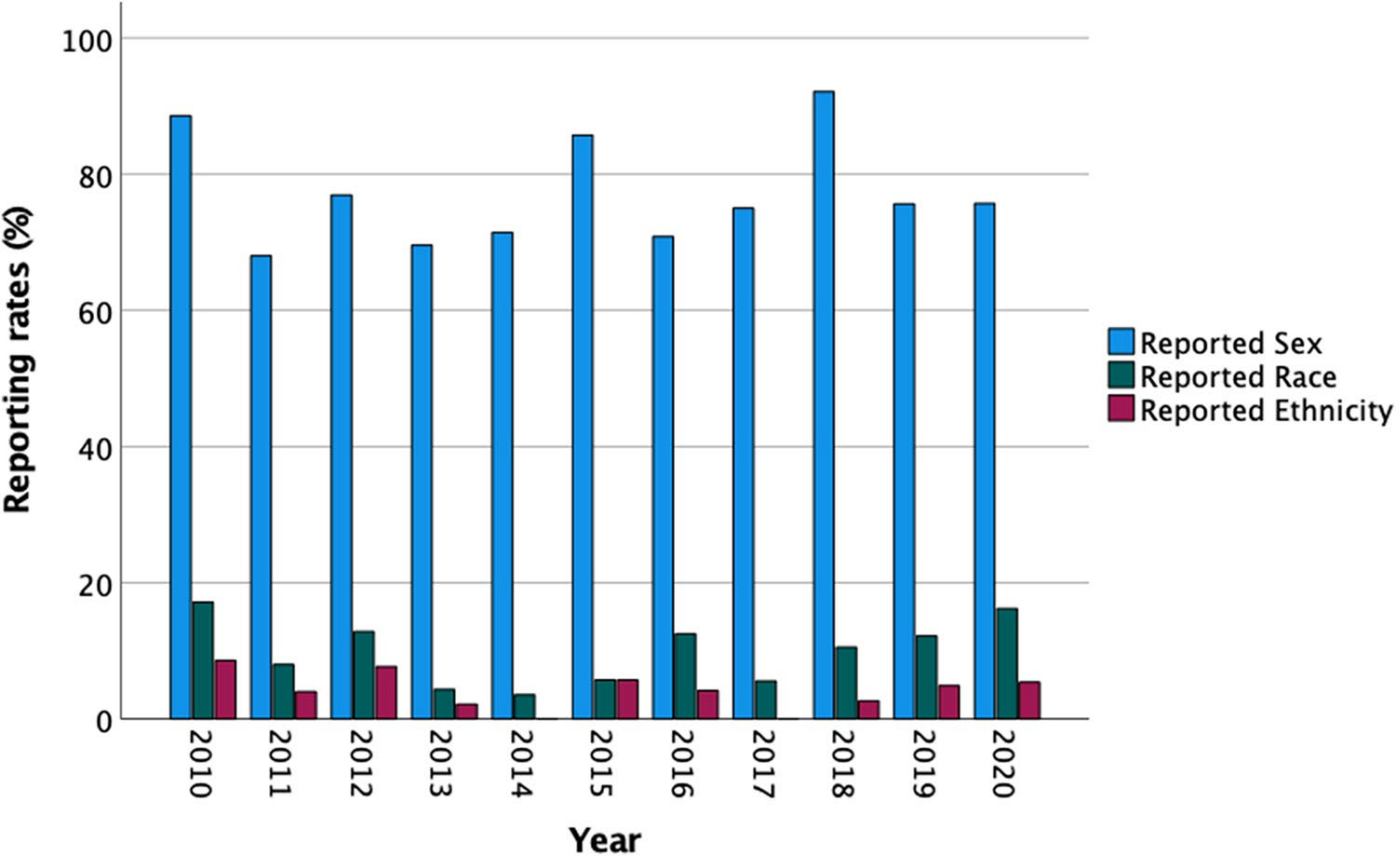
Demographic reporting rates in neuroimaging studies over time. Reporting rates, calculated as the number of articles with reported demographics out of the total number of included articles per year, are shown for sex, race, and ethnicity. No apparent trends over time were observed. Article counts for each year are provided in Supplementary Table 2

Of the 315 studies reporting biological sex (77% of all included articles), males and females were represented nearly equally (51% males, 49% females) and no apparent trends were observed as a function of time (Fig. 2, Supplementary Table 2). In addition to inclusion of both biological sexes, the NIH implemented a related policy in 2016 requiring the use of sex as a biological variable given documented differences in anatomy, physiology, disease presentation, and patient outcomes (Bonkhoff et al. 2021; Liu et al. 2020; Wright et al. 2020). Utilizing 488 subjects in the HCP database, Liu et al. observed significant differences in brain structure. In some regions, gray matter volume was larger in females (insula, orbitofrontal cortex) and in others larger in males (fusiform gyrus, amygdala) (Liu et al. 2020). Scrutinio et al. observed a decreased risk of mortality among female compared to male stroke victims when controlling for age (Scrutinio et al. 2020). Gilsanz et al. observed unique risk factors for dementia in females not observed in males such as mid-adulthood hypertension (Gilsanz et al. 2017). While inclusion of female subjects and animal models has improved in some fields (Beery and Zucker 2011; Woitowich et al. 2020), few studies include sex as an analytical variable and many still report higher inclusion of male compared to female animal models (Vognsen et al. 2017; Mamlouk et al. 2020). As highlighted in a recent study by Garcia-Siffuentes and Maney, a minority of biological studies properly assess the impact of biological sex (Garcia-Sifuentes and Maney 2021), emphasizing the need for both inclusion and analysis of sex-related disease factors.

While we denote the number of male and female participants as biological sex, several studies used the term gender when reporting the number of male and female subjects (Iltis et al. 2010; Awate et al. 2010) and others report these same demographics as sex (Tseng, et al. 2020; Cheong et al. 2017). It was unclear if biological sex or gender identity was reported by participants. Previous work provides evidence for brain structural differences between transgender and cisgender individuals (Flint et al. 2020). As an example, in a study where biological sex was classified based on brain volumes using a support vector machine, researchers were able to correctly identify cisgender women (95%) but the classifier was less accurate for transgender women (56%) (Flint et al. 2020). These data support the importance of consistent and transparent terminology in reporting as well as further examination of the effects of both sex and gender.

## Race and ethnicity are underreported across neuroimaging studies

Racial categories used in this study include American Indian or Alaska Native, Black or African American, Asian or Pacific Islander, White, more than one race, and other race. Ethnicity was defined as Hispanic or Latino, Non-Hispanic or Latino, and other ethnicity, consistent with classifications used by the NIH and U.S. Census. As the extent and method of racial reporting varied substantially between studies, we indicate a racial group of other race to denote participants who were not specified. For example, one article reported race as 70% Caucasian but did not report the race of the remaining 30% of participants and we noted these as other race (Supplemental Tables 1 and 3) (Cohen-Gilbert et al. 2015). Some articles also use the term other, explicitly, to denote race of participants. Lack of uniform reporting standards remains a challenge, particularly in large epidemiological studies or meta-analyses. In a recent systematic review and meta-analysis comparing health outcomes as a function of ethnicity, researchers were also required to develop a system to combine racial and ethnic categories to facilitate analysis due to inconsistencies in reporting (Sze et al. 2020).

In comparison to sex, which was reported in the majority of included articles, 41 (10%) of all included articles reported race and 17 (4%) reported ethnicity. Similar to reporting of sex, we observed no apparent trends as a function of time (Fig. 2, Supplementary Table 2). For all articles reporting race, ethnicity, or both, sex of participants was also reported, suggesting reporting one demographic trait may increase the likelihood of reporting others. For articles reporting race (*n* = 41), a minority also reported ethnicity (*n* = 16, 38% of articles reporting race); however, the reporting rate was higher than the overall reporting rate for ethnicity across all studies (38% of articles reporting race compared to 4% of all included articles).

**Fig. 3:**
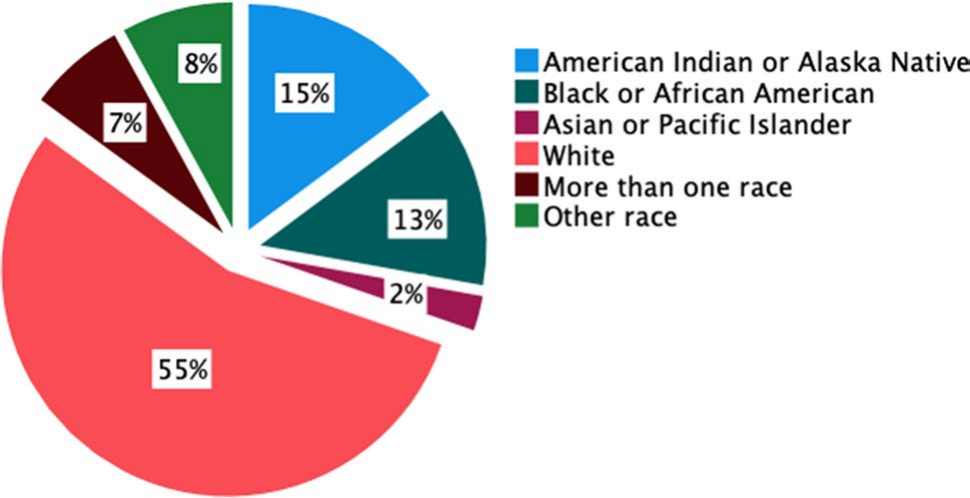
Racial demographics across all articles reporting race. For articles reporting race (*n* = 41, 10% of all included articles), the most well-represented race was White, followed by American Indian or Alaska Native, which was largely attributed to a single study focused on this racial group. The term other race is used to denote racial categories not otherwise specified in the articles, or articles that use this term explicitly in their reporting (see Supplementary Methods)

The racial diversity of reported participants is shown in Fig. 3 and Supplementary Table 3, with the majority of reported participants classified as White (*n* = 2,924, 55% of participants for which race was reported). While representation of some racial groups was consistent with current U.S. demographics as reported in the 2020 U.S. Census, a few exceptions were noted. The first was the underrepresentation of Asian participants (*n* = 133, 2% of participants for which race was reported), consistent with previous reports that individuals who identify as Asian or Asian American are largely underrepresented in biomedical research (Reihl et al. 2022). The second was a higher percentage of American Indian or Alaska Native participants (*n* = 792, 15% of participants for which race was reported) relative to the U.S. Census (1% of the U.S. population). This was largely driven by a single study published in 2017 using data from the Cerebrovascular Disease and its Consequences in American Indians (CDCAI) study, which included 1,033 individuals from 11 Native American tribes in the U.S. (Suchy-Dicey et al. 2017). This study accounted for 99% of the reported American Indian or Alaska Native participants across all included studies. In the absence of this single study, American Indian or Alaska Native participants comprised 0.4% of participants for which race was reported. Importantly, the demographics of participants across included neuroimaging studies cannot be fully determined given the overall lack of reporting. A total of 36,312 participants were represented in the included articles, yet race was only reported for 5,342 participants (15% of the total participants). A previous study by Fansiwala et al. reported among 393 clinical trials examining neurological diseases (stroke, epilepsy, and Alzheimer’s disease), 20% of articles reported race (Fansiwala et al. 2017), consistent with what we observed for neuroimaging studies.

## Challenges in reporting race and ethnicity in biomedical research

Race and ethnicity have both been associated with unique phenotypic traits. For example, studies have observed significant differences in the corpus callosum between sub-populations of the Middle East (Allouh et al. 2020; Hosseini et al. 2018). In a study comparing brain volumes from healthy White (*n* = 44) and Black or African American (*n* = 25) individuals, volumetric differences were observed in the left orbitofrontal cortex (Isamah et al. 2010). Furthermore, neuroanatomical differences in gyri located in the frontal, parietal, and temporal lobes were identified between English-speaking White and Chinese-speaking Asian individuals (Kochunov et al. 2003).

While genetic differences exist between individuals, these are generally not well-delineated between racial groups. Individuals may, for example, identify as Black or African American but have more similar genetic ancestry to a White individual of European descent than an individual from a different region in Africa (Bryc et al. 2015). Twin studies further exemplify the complication of assigning differences according to race. Burghy et al. examined the role of genetics and environmental influences in 26 monozygotic twins and reported cortisol levels during adolescence were an indicator of different environmental influences (stress) and predicted differences in brain anatomy and function over time (Burghy et al. 2016). Higher cortisol levels measured during childhood were associated with lower resting state connectivity later in life compared to a twin with lower cortisol levels, supporting the established theory that individual experiences may be more important than genetic or other hereditary traits (Burghy et al. 2016).

Race may be best defined as a social construct and more appropriately used as a surrogate for other factors such as stress (Geronimus 1992), diet (Leung and Tester 2019), or access to health care (Weissman et al. 2018). The challenge lies in defining the appropriate terminology and classification of racial groups (Spector et al. 2016). In a study comparing the accuracy of self-identified race or ethnicity to genetically determined ancestry, self-identified versus genetically-determined ancestry differed for nearly 10% of participants (Spector et al. 2016). In the absence of a genetic explanation for observed differences in biology or disease manifestation, sociological components may directly or indirectly impact health and disease. For instance, race can impact quality and extent of healthcare due to stigmatization, bias, or other racial inequities (Wiens et al. 2020). For infants born to Black mothers, evidence supports a survival advantage of infants born to teen compared to older mothers (Geronimus 1992). These data reflect what is known as the weathering hypothesis, or declining health of African American women with age due to chronic stress, derived from socioeconomic disadvantages that are correlated with race (Geronimus 1992). In a systematic review by Forde et al., 37 out of 41 articles reported evidence in support of the weathering hypothesis and identified associated health disparities such as birth weight, mortality, and blood pressure (Forde et al. 2019). While outside the scope of this review, disentangling the effects of race and other social constructs is an important future goal.

Many well-established brain atlases (e.g., International Consortium for Brain Mapping atlas) utilize primarily White participants (Mazziotta et al. 2001; Wu et al. 2021). Recent work has attempted to ameliorate differences assigned to race and ethnicity by developing brain atlases and databases using participants of a single racial or ethnic group (Tang et al. 2010; Valdes-Sosa et al. 2021). For example, some recent atlases have focused their enrollment on non-White racial groups such as the Chinese brain atlas and the Indian brain atlas (Tang et al. 2010; Sivaswamy et al. 2019). The goal of these atlases is to develop data sources that account for documented neuroanatomical differences in the brain across race and ethnicity. Additional efforts such as the Cuban Human Brain Mapping Project provide further support for trends of focused recruitment of underrepresented racial and ethnic groups (Valdes-Sosa et al. 2021). Beyond the challenges of using these atlases in a diverse sample as generally a single atlas is used for all participants in a study, there is also the ethical concern of identifying and classifying anatomy solely on the basis of race or ethnicity. Further consideration is warranted.

## Reporting varies according to disease of focus and age of participants

**Fig. 4:**
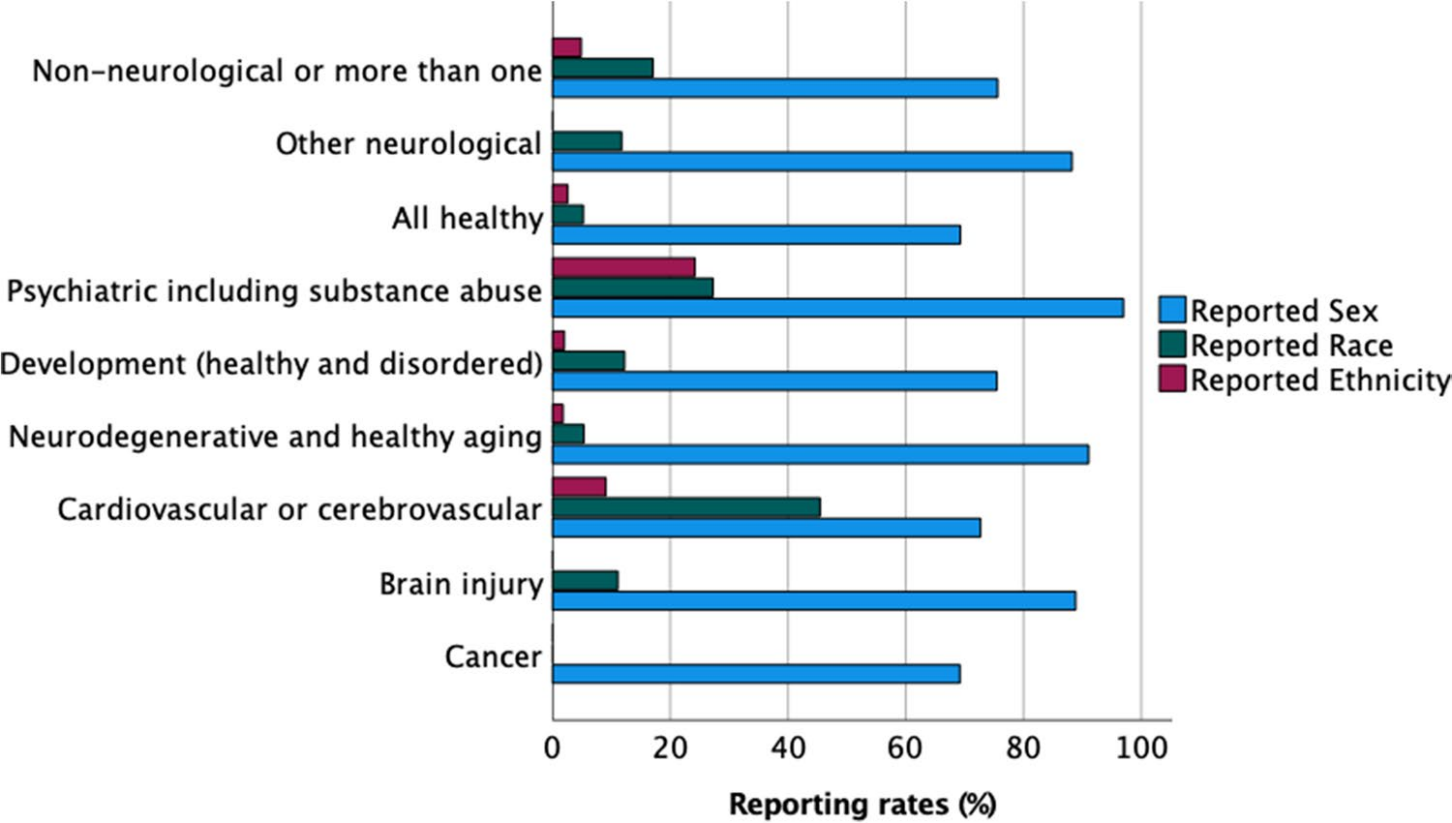
Demographic reporting as a function of disease. Reporting rates for sex, race, and ethnicity varied depending on the study cohorts. Biological sex was reported at the highest rates in articles focused on psychiatric disease and lowest in studies of all healthy subjects. The highest reporting of race was observed in studies focused on cardiovascular or cerebrovascular disease. Articles focused on cancer did not report race or ethnicity for any participants. Within studies reporting race, the majority of participants were White with the exception of articles including all healthy participants (largely driven by a single study with high inclusion of mixed race individuals)

To identify additional reporting trends as a function of cohort, articles were classified according to disease of focus and age range (see Supplemental Methods). Among all 408 articles, the most represented cohort was all healthy participants (*n* = 153 articles, 38% of all included articles), followed by neurodegenerative and healthy aging (*n* = 56, 14% of all included articles), and development (healthy and disordered) (*n* = 49, 12% of all included articles). Biological sex was reported at the highest rate in studies focused on psychiatric disease (*n* = 32, 97% reporting rate), followed by neurodegenerative and healthy aging (*n* = 51, 91% reporting rate), and brain injury (*n* = 8, 89% reporting rate) (Fig. 4). In comparison, the lowest reporting of sex was observed in studies of all healthy cohorts (*n* = 106, 69% of articles classified as healthy reported sex). Of these, a total of 7,196 healthy participants were represented and male and female subjects were represented nearly equally (*n* = 3,617 males and *n* = 3,579 females). Further analysis revealed the largest imbalance between male and female participants were reported in studies focused on brain injury cohort. Of a total of 238 participants, males comprised the majority (*n* = 171, 72%) compared to females (*n* = 67, 28%). This is consistent with the underrepresentation of female subjects in brain injury studies, despite the prevalence of traumatic brain injury in females which account for ~ 41% of brain injury-related medical visits in the U.S. (Biegon 2021).

The highest rates of reporting race were observed in studies focused on cardiovascular or cerebrovascular disease (*n* = 5, 45% reporting rate), followed by psychiatric disease (*n* = 9, 27% reporting rate), and non-neurological or more than one disease (*n* = 7, 17% reporting rate) (Fig. 4). None of the studies focused on cancer (*n* = 39) reported race. Low reporting rates for race were also observed for studies of neurodegenerative disease and healthy aging (*n* = 3, 5% reporting rate) and all healthy participants (*n* = 8, 5% reporting rate).

The reported racial diversity also varied according to disease type. We observed White participants comprised the majority of studies across multiple disease groups (brain injury, cardiovascular or cerebrovascular, neurodegenerative and healthy aging, developmental (healthy and disordered), psychiatric, and other neurological disorders). In studies focused on brain injury, White participants represented 90% of participants. White participants were represented least in studies of all healthy participants (32%). The highest inclusion rates of Black or African American participants were in studies of neurodegenerative disease and healthy aging (38% Black or African American participants) and the lowest incidence in studies of cardiovascular or cerebrovascular disease (0.2% Black of African American). These data demonstrate within disease cohorts, reported racial diversity can vary; however, the demographics represent a small fraction of the participants included across all studies and comparisons to disease prevalence are difficult.

**Fig. 5:**
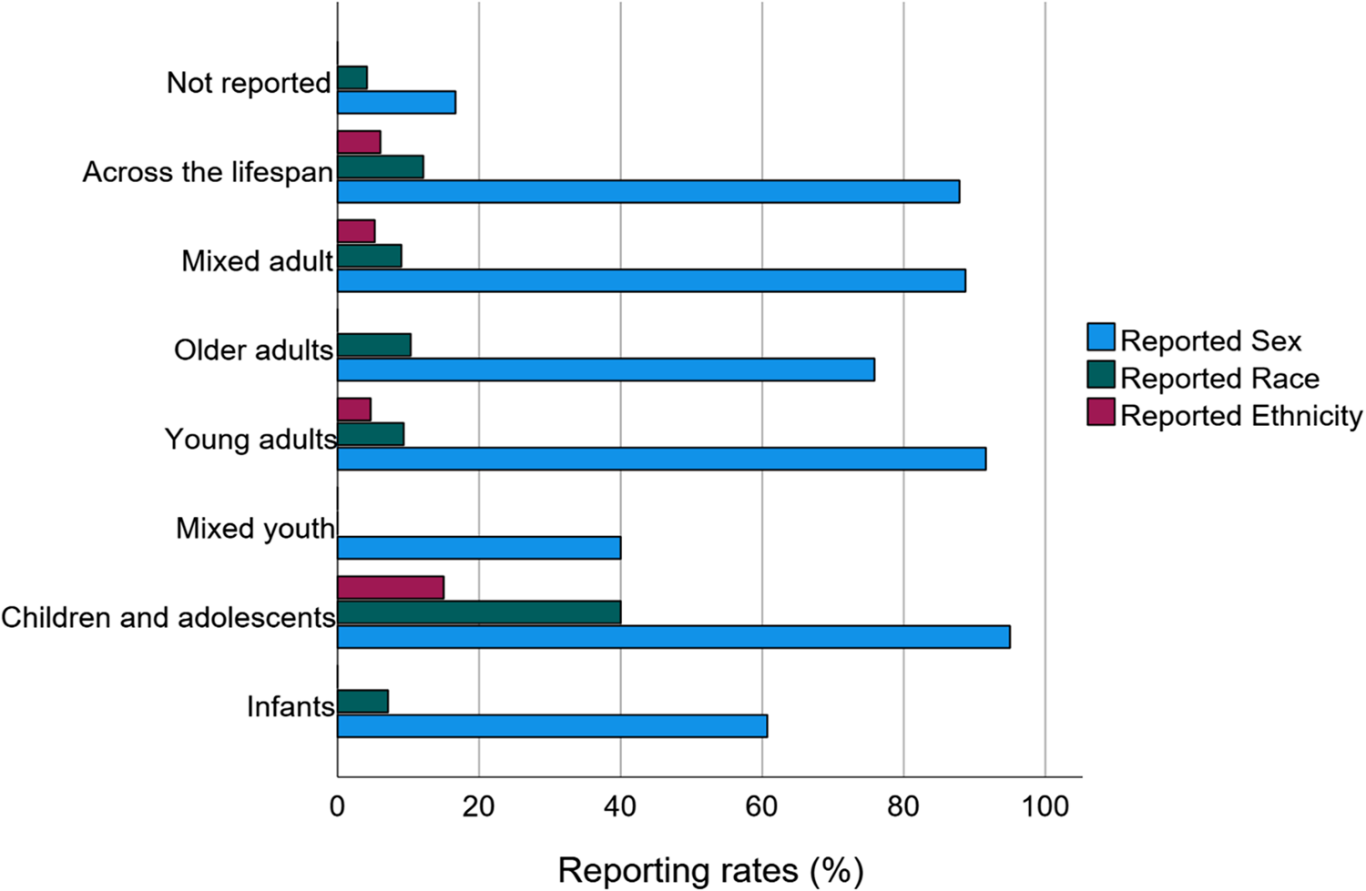
Demographic reporting as a function of subject age. Reporting rates for sex, race, and ethnicity varied depending on the age of included participants. Age of participants was categorized as infant (0–2 years old), children and adolescent (3–18 years old), mixed youth (0–18 years old), young adult (18–49 years old), older adult (≥ 49 years old), mixed adult (≥ 18 years old), across the lifespan (both youth and adults), and not reported. Biological sex was reported highest in articles including children and adolescents and lowest in studies of mixed youth. Race was also reported highest in studies of children and adolescents and was not reported in studies of mixed youth. Ethnicity was not reported in studies of infants, mixed youth, older adults, or in studies without reported age of participants

Rates of demographic reporting also varied with the age group of included participants (Fig. 5). The majority of articles included young and older adult participants ≥ 18 years old (*n* = 133, 33% of all included articles), followed by studies of young adults between the ages of 18–49 years old (*n* = 107, 26% of all included articles). Studies of participants 0–18 years old represented the fewest studies (*n* = 10, 2% of all articles), followed by children and adolescents (3–18 years old; *n* = 20, 5% of all articles). Interestingly, reporting rates for sex were highest among studies that utilized children and adolescents (*n* = 19, 95% reporting rate for sex), and lowest among studies that utilized mixed youth cohorts (including infants as well as children and/or adolescents; *n* = 4, 40% reporting rate for sex). Race was reported at the highest incidence in studies that utilized children and adolescents (*n* = 8, 40% reporting rate for race), and lowest among studies that did not report age (*n* = 2, 4% reporting rate for race).

Ganguli et al. reported females (*n* = 535, 58%) were more willing than males (*n* = 380, 42%) to volunteer for MRI research (Ganguli et al. 2015), and some of the equal representation of males and females we observed across all included articles is likely due to a high sampling of healthy and young adult participants across neuroimaging studies. This is, in part, reflected by the data as reported sex in studies of young adults 18–49 years old were 50% male (*n* = 2,793) and 50% female (*n* = 2,779), with a 92% reporting rate for sex in articles that included this age cohort.

## Reporting in NIH funded studies

A total of 352 articles (86% of all included articles) reported funding or a portion of funding from the NIH. Reporting rates of sex, ethnicity, and race were all highest in studies reporting NIH funding. Of those receiving NIH funding, 279 (79% of all articles reporting NIH funding) reported sex, 37 (11%) reported race, and 16 (5%) reported ethnicity. In comparison, for articles that did not report NIH funding, 36 (64%) reported sex, 4 (7%) reported race, and 1 (2%) reported ethnicity. Interestingly, a review by Mamlouk et al. demonstrated biological sex in neuroscience research was reported at similar rates regardless of NIH funding (Mamlouk et al. 2020). Reported racial diversity of participants in studies with reported NIH funding was comparable to the overall racial distribution (Fig. 3) and included American Indian or Alaska Native (15%), Black or African American (12%), Asian or Pacific Islander (3%), White (56%), more than one race (7%), and other race (8%). The NIH Revitalization Act mandates the inclusion of women and minorities, but does not provide specific guidelines for included participants, particularly when inclusion or exclusion of one or more race can be scientifically justified. It is possible some of the distribution may be justified by the disease of interest, demographics of the region, or other factors.

## Trends as a function of publisher

While funding agencies regulate some aspects of inclusion and reporting, a bigger influence may be the publishers themselves (see Supplemental Methods). The most well-represented publisher was Elsevier (*n* = 129 articles, 32% of all included articles), followed by Wiley (*n* = 100, 25% of all included articles). Among articles published by Elsevier and Wiley, we observed similar reporting rates for sex (78% for both) and ethnicity (5% Elsevier; 3% Wiley). Reporting rates for race were higher in articles published by Elsevier (11%) compared to Wiley (5%). The highest rates of reporting sex were observed in articles published by Nature (*n* = 13, 87% reporting rate) and professional societies or associations (*n* = 58, 79% reporting rate). The highest rates of reporting race were observed in articles published by Nature (*n* = 5, 33% reporting rate) and Springer (*n* = 8, 29% reporting rate). Reporting rates were similar for ethnicity, with the highest rates observed in articles published by Nature (*n* = 3, 20% reporting rate) and Springer (3, 11% reporting rate). The lowest reporting rate for sex was observed among articles published by Springer (68% reporting rate) followed by PLoS (69% reporting rate). The lowest reporting rates for race and ethnicity were observed in articles published by PLoS (0% for both race and ethnicity). Ethnicity was also not reported in any studies published by professional societies or associations. A recent letter published in *JAMA Psychiatry* suggests structural racism, indicated by underrepresentation of diverse racial and ethnic groups at the editorial level, may also influence journal content (Shim et al. 2021). In a survey of psychiatry and neuroscience journals, 283 journal editors self-reported their demographics, including race and ethnicity. Of these, approximately 27% identified as Black, 10% as Asian, 17% as Hispanic/Latinx, and 48% as White. Among those who reported serving as editor in chief, approximately 3% identified as Black, 0% as Asian or Indigenous, 6% as Hispanic/Latinx, and 84% as White. While the sample was self-selective, the results further emphasize the presence of structural racism which may, indirectly or directly, influence demographic reporting as a result of a complex interaction of factors, ranging from researchers to publishers.

## Barriers to reporting and inclusion

Barriers to diversity in neuroscience and neuroimaging research are vast. From interviews with patients, physicians, investigators, and study coordinators, it has been suggested diversity in clinical trials is limited primarily by time and resource constraints, particularly for Asian, Black, and Hispanic participants (Clark et al. 2019). Location can be particularly challenging for individuals residing in rural areas (Feyman et al. 2020). In a study of participation in clinical trials, a positive correlation was observed between population size and total number of trial participants, suggesting more urbanized areas may have a greater influence on diversity in clinical trials (Feyman et al. 2020). While stratification of our data by study site or state was not possible given several multi-site studies and lack of detailed recruiting methods in many articles, this is an important consideration for future work. An additional barrier is differences in spoken and/or written language between researchers and participants (Durant et al. 2014). In interviews of 91 individuals in various roles in clinical trials, Durant et al. reported language was a barrier for the majority of participants (> 50%) (Durant et al. 2014).

Financial difficulties can also pose a barrier to diversity in clinical trials and health research. In a systematic review conducted by George et al., 44 articles surveyed barriers reported by racial and ethnic minorities to participating in research (George et al. 2014). Nearly half (45%) reported difficulties due to time and financial constraints (George et al. 2014). Financial considerations also affect researchers and the studies themselves. With limited funding budgets, considerations of diversity may be outweighed by the need for reduced variability in a limited cohort size. This is particularly challenging for neuroimaging studies where study costs can be high.

Furthermore, medical mistrust as a result of historical abuses towards minority groups is an important consideration (Yancey et al. 2006). Studies of HIV treatment provide evidence that different racial groups experience varying levels of medical trust that may impact willingness to seek treatment (Meyers-Pantele et al. 2021). Individuals often attribute medical mistrust to previous challenges in medical settings (Hall et al. 2022). In a study of 143 Black individuals who received medical treatment, a majority (79%) reported racial discrimination (Hall et al. 2022). In research studies, specifically, historic examples of unethical research practices, such as the Tuskegee syphilis study and experiments performed on inmates in World War II Nazi concentration camps, are cited as reasons for medical mistrust (Algahtani et al. 2018). Given abuse of minorities in research and current bias in the U.S. healthcare system against non-White and other marginalized individuals, it is reasonable to expect not all participants in research studies may be willing to disclose their gender identity, race, or other demographic factors. Balancing the necessary right of individuals to refuse participation and reporting with the importance of effective diagnosis, prognosis, and treatment of diverse populations is an outstanding challenge.

## Limitations, outlook, and future recommendations

Given our initial focus on the NIH Revitalization Act which applies to NIH funded studies, we limited inclusion to research performed in the U.S. It is expected demographics will vary depending on the local population and recruitment pool. This is particularly important when considering large database efforts, particularly cross-continental studies with recruiting in multiple countries or in different regions of large countries, including the U.S. Our own reporting was also limited by how demographics were reported in the articles themselves, and some assumptions were made particularly when reporting was opaque. Sex and gender were used interchangeably in multiple articles, and the methods for reporting race and ethnicity (e.g., Hispanic was often used as a classification for race) varied substantially between studies. Furthermore, demographics for a small subset of participants may be redundant as several articles with reported sex or racial demographics utilized the same databases (e.g., HCP). While our goal was to characterize demographics of participants in neuroimaging research, we only included those utilizing MR methods to limit the scope of our review to a reasonable number of articles. Finally, given the search criteria (“human brain MR”, see Supplementary Methods), it is possible the articles utilized in this review are a subset of those meeting inclusion criteria. We acknowledge this limitation; however, the systematic nature of the review supports an unbiased and likely representative sample.

Future efforts should be made to report both biological sex and gender identity of participants to facilitate a deeper understanding of differences on the basis of both. While sex is often recorded by researchers, recording both sex and gender is a simple next step to facilitate further research in this area. For race and ethnicity, challenges exist for unifying definitions as guidelines differ across funding agencies, journals, and countries. Race should also be considered as a combination of both social and biological factors moving forward. More effective metrics may include socioeconomic status, zip code of domicile, or access to resources. Considering the limited evidence for the role of genetics on observed differences between racial groups (Bryc et al. 2015; Spector, et al. 2016), and the subsequent literature that details environmental and social influences on race (Geronimus 1992), it is prudent to develop relevant metrics, beyond phenotypic traits, to ensure broad applicability of research results. The intersection of multiple demographic and social factors, particularly for groups historically underrepresented in research, is also an important consideration. Targeted recruitment of a racial or ethnic group, for example, must also consider diversity within the group across other factors such as sex and gender, age, and socioeconomic status. This intersectionality may be particularly germane for research conducted in countries with different demographic composition than the U.S., i.e., more or less racial and ethnic diversity.

An outstanding question in biomedical research is the inclusion of equal numbers of participants across all racial groups or representation based on disease prevalence. This is a challenge particularly for diseases with different clinical presentation across demographics groups (e.g., autism spectrum disorder presents differently in males compared to females) and is an important consideration for future work. Given underreporting and limited research for most diseases regarding demographic differences in presentation and progression, a concerted effort to understand and quantify disease effects as a function of race, sex, and age, among others, is imperative, particularly as advanced neuroimaging methods continue to emerge in translational research (Ekici et al. 2022; Fleischer et al. 2017; Sung et al. 2021; Port 2018; Bharti et al. 2019; Kohoutova et al. 2020). While some researchers focus on populations most affected by a disease, it is often difficult to assess the full impact of a disease on a demographic group given the lack of diversity across participants included in biomedical research and clinical trials. Challenges remain with small sample sizes and heterogeneous populations, and transparent reporting may be one approach to address the lack of diversity. Open access efforts, large databases, and projects such as the NIH brain research through advancing innovative neurotechnologies (BRAIN) Initiative and HCP, complemented by data sharing, meta-analyses, pooled studies, and other large-scale studies may facilitate analysis that considers demographic variables analytically and holistically. Collaborative multi-institution and multi-national studies may be crucial to this effort, particularly for research with high associated costs including neuroimaging (Grant and Chamberlain 2018). It will also be important for large databases to include comprehensive demographic data, as much of this data is restricted or unavailable at the individual participant level. A recent perspective by Shansky and Murphy discuss the large-scale shift required to consider sex as a biological variable in neuroscience research (Shansky and Murphy 2021), and much of their discussion is broadly applicable to other demographic considerations.

Multiple fields within the scientific community, in response to growing acknowledgment of variation in brain function and disease across groups, are advocating for more diverse populations in research (Landry et al. 2018) as well as more transparent reporting (Landis et al. 2012; Collins and Tabak 2014). Human physiology and disease must be studied across a diverse population to fully understand pathology and progression as a function of demographic and related factors. Indeed, a key goal of biomedical research is broad generalizability and translation, and the fact remains that diversity and transparent reporting are still lacking in neuroimaging and biomedical research. We provide a few recommendations towards the goal of expanded impact and widespread applicability. First, demographic descriptors including sex, gender, race, ethnicity, and age must be reported for all human subjects in peer-reviewed primary research articles. Several publishers and consortiums provide guidance on reporting such as the International Committee of Medical Journal Editors (http://www.icmje.org/recommendations/), but more specificity may be required to ensure transparent reporting. Furthermore, while many journals require reporting of sex and race for all participants, age ranges or ‘matched’ controls are often sufficient and this lack of specificity hinders analysis that considers these factors explicitly. Second, statistical analysis particularly in large imaging studies and/or meta-analyses must consider factors beyond biological sex or self-identified race. Accounting for socioeconomic factors, healthcare access, country of birth or residence, and gender, among others, may provide a more nuanced view of brain function and disease manifestation in diverse communities and populations. Third, open access to demographic data, after deidentification, should be a major goal of scientific publishers, data repositories, and funding agencies. Lastly and undoubtedly the most challenging, a shift in the conduct of biomedical research will be required. This may involve further development and funding of research centers lead by large teams rather than a primary focus on individual investigator funding, particularly in the study of prevalent diseases or healthy individuals (e.g., NIH All of Us Research Program, UK Biobank). Another approach may be the collective effort to overrecruit populations historically underrepresented in neuroimaging and disease studies. While this is a multi-faceted and long-term challenge, lack of diverse representation, particularly with regard to race and ethnicity, nearly 30 years after the NIH Revitalization Act was passed necessitates a larger conversation and dedicated effort.

As the U.S. and the world at large become more diverse, particularly in urban areas where the majority of neuroimaging research is performed, a concerted and conscious effort by researchers, funding agencies, publishers, and institutions is needed to ensure broadly applicable and translatable results and long-lasting impact of biomedical and neuroscience research.

## Supplementary Information

Below is the link to the electronic supplementary material.

Supplementary file1 (XLSX 56.4 KB)

Supplementary file2 (XLSX 9.37 KB)

Supplementary file3 (XLSX 10 KB)

Supplementary file4 (PDF 181 KB)

Supplementary file5 (PDF 116 KB)

## Data Availability

All data collected in the systematic review are included in Supplementary Table 1.

## Code Availability

N/A.
