# Supplementary material for: Demographic reporting across a decade of neuroimaging: a systematic review
Source: Brain Imaging Behav. 2022 Sep 17;16(6):2785–96. doi: 10.1007/s11682-022-00724-8 (PMC9712398; doi:10.1007/s11682-022-00724-8)
Supplement: Supplementary file 4 — Supplementary file4 (PDF 181 KB) [file 11682_2022_724_MOESM4_ESM.pdf]

Supplementary Information

Demographic reporting across a decade of neuroimaging: a systematic review

Elijah Sterling[1], Hannah Pearl[2], Zexuan Liu[3], Jason W. Allen[3,4,5], and Candace C. Fleischer[3,4]

[1]College of Arts and Sciences, Emory University, Atlanta, Georgia, USA
[2]School of Arts and Sciences, Tufts University, Medford, Massachusetts, USA
[3]Department of Biomedical Engineering, Georgia Institute of Technology and Emory University, Atlanta, Georgia, USA
[4]Department of Radiology and Imaging Sciences, Emory University School of Medicine, Atlanta, Georgia, USA
[5]Department of Neurology, Emory University School of Medicine, Atlanta, Georgia, USA

Key Words: Demographic reporting, NIH Revitalization Act of 1993, neuroimaging, MRI

To whom correspondence should be addressed:

Candace C. Fleischer, PhD

Wesley Woods Health Center

1841 Clifton Rd NE

Atlanta, GA 30329

404-712-1020

candace.fleischer@emory.edu

Contents:

Supplementary Methods

Supplementary Tables 1-3

Appendix 1. PRISMA 2020 Checklist and Abstracts Checklist

Supplementary Methods

A systematic review was conducted using the PRISMA guidelines (http://www.prisma-statement.org/, Appendix 1). This review was not registered; all protocol details are provided herein. Initial search criteria using the Web of Science™ search engine were article topic of "human brain MR", the paper was written in English, the document type was article, and the final version of the article was published between 2010-2020, inclusive. The abbreviation MR was used rather than "magnetic resonance" as many MR journals do not require the full spelling of this acronym and would likely result in exclusion of relevant MR-focused articles. The word *human* was used to limit the number of pre-clinical studies included in the initial search and reduce the number of articles to a reasonable number ("human brain MR" identified 3,458 articles compared to "brain MR" which included 15,538 articles). We acknowledge some studies use the terms volunteer or patient, but not human, and were excluded as a result. Data collection was performed between June 2021 and November 2021.

Exclusion criteria were the article was not a primary research article (e.g., review), human brain MR data was not acquired, <10 participants were included, MR data was acquired outside the United States (U.S.), and the journal did not meet a quality control check. To determine whether a study was conducted in the U.S., a combination of the imaging location or center, name of the Institutional Review Board, and affiliation of the authors was used based on information reported. Journal quality was assessed using the Think-Check-Submit method (https://thinkchecksubmit.org). If two or more criteria could not be met, the article was excluded.

A flow chart for article selection and exclusion is shown in Figure 1. Although a single article may have met multiple exclusion criteria, articles were excluded and tallied in the order listed in Figure 1. Articles meeting initial search criteria were reviewed by two independent researchers (one primary and two secondary). The senior investigator not involved in data collection compared the independent results for each manuscript. Discrepancies were resolved through a further review of the article by the two initial researchers and the senior investigator to reach consensus. Of note, given nuances in the Think-Check-Submit method, all exclusion based on journal quality was performed by the senior investigator. All included articles and associated data are reported in Supplemental Table 1.

Biological sex was reported as male or female, indicated by either biological sex or gender in the article. Race was categorized as American Indian or Alaska Native, Black or African

American, Asian or Pacific Islander, White, more than one race, and other race. The categories for ethnicity included Hispanic or Latino, Non-Hispanic or Latino, and other ethnicity. Other was used for vague reporting, e.g., race or ethnicity was reported as “undisclosed,” or some participants were listed as non-White without any additional information. Some articles reported demographics for a subset of the participants rather than the total, and some used the term other race, explicitly, as a demographic classification. All reported demographics were recorded, even if incomplete within a study, and included in our final analysis. Additionally, some studies classified Hispanic as a race, and these were reported as ethnicity for our purposes.

Disease was classified into one of the following categories: cancer, brain injury, cardiovascular or cerebrovascular, neurodegenerative and healthy aging, development (healthy and disordered), psychiatric including substance abuse, all healthy, other neurological or central nervous system (CNS) disease, and non-neurological or more than one disease. Age was classified based on the reported age range of all participants. If an article reported a mean and standard deviation but no range, the age range was estimated. Age classification included infants (0-2 years old), children + adolescents (3-18 years old), mixed youth (0-18 years old), young adults (18-49 years old), older adults (≥49 years old), mixed adults (≥18 with no upper limit), across the lifespan (infants and/or youth and adults), and not reported. Finally, articles were categorized based on the publisher, and categories included Nature, Elsevier, Springer, Wiley, professional society or association, academic or university publisher, PLoS, and other (publishers represented by ≤15 articles each). While Nature is now Springer Nature, the publishing process differs between the Nature family of journals and Springer, so these were differentiated in analysis. All analysis and data presentation were facilitated with IBM SPSS (v28). All percentage values within the text were rounded to the nearest whole percentage for simplicity, resulting in some total values not equal to 100%. The reader is directed to the raw data in Supplementary Table 1 for further information.

Supplementary Tables

Supplementary Table 1. Citations and data for all included articles. Reporting is denoted as yes (1) or no (0). For articles with reported demographics, numbers are included for sex, race, and ethnicity. (Sterling_Supplemental_Table_1.xlsx)

Supplementary Table 2. Rates of demographic reporting for all included articles from each year. The number of articles reporting each demographic (N) and percentages (calculated using the number of total articles) for each year are shown. (Sterling_Supplemental_Table_2.xlsx)

Supplementary Table 3. Reported demographics for articles with reported sex, race, and/or ethnicity for each year. For studies reporting demographic information (Supplementary Table 2), percentages for inclusion (calculated using the number of total participants) are shown for each year and across all studies. Percentages ≤0.05% were rounded to 0%. (Sterling_Supplemental_Table_3.xlsx)
